# Supplementary material for: Structural specificities of cell surface β-glucan polysaccharides determine commensal yeast mediated immuno-modulatory activities
Source: Nat Commun. 2021 Jun 14;12:3611. doi: 10.1038/s41467-021-23929-9 (PMC8203763; doi:10.1038/s41467-021-23929-9)
Supplement: Supplementary file 1 — Supplementary information [file 41467_2021_23929_MOESM1_ESM.pdf]

Supplementary information

Structural specificities of cell surface β-glucan polysaccharides determine commensal yeast mediated immuno-modulatory activities

Lee et al.

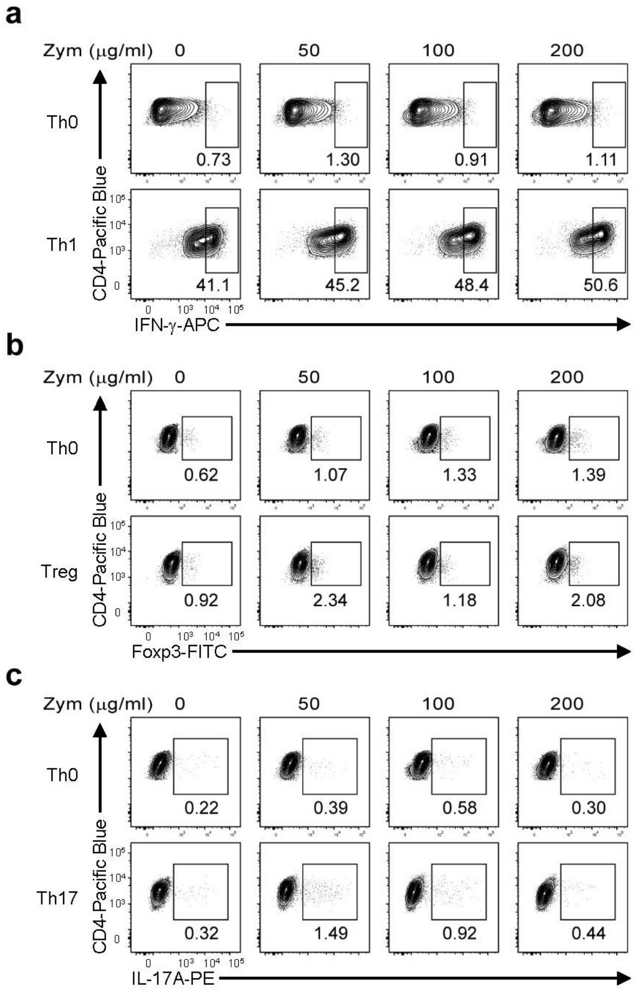

Figure S1. Effect of zymosan on T cell differentiation. (**a-c**) Splenic CD11c$^+$ DCs were stimulated with zymosan and cultured with naïve CD4$^+$ T cells either with Th0 condition, or in suboptimal Th1 (a), Treg (b) and Th17 (c) differentiation conditions *in vitro*. Representative flow cytometric plots of differentiated cells under indicated conditions are shown.

a

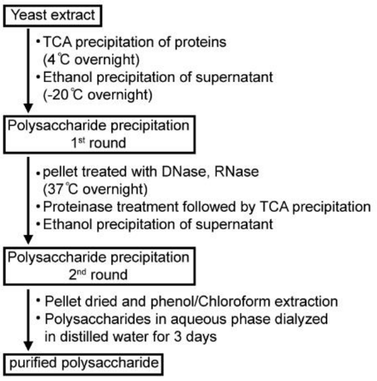

d

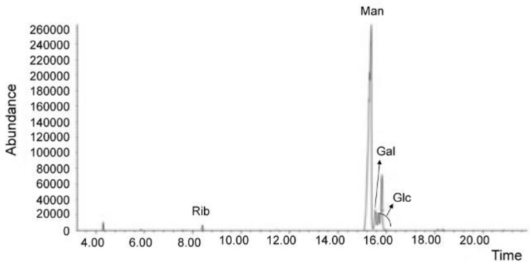

	Mannose	Galactose	Glucose
MGCP	5.4	0.3	1

b

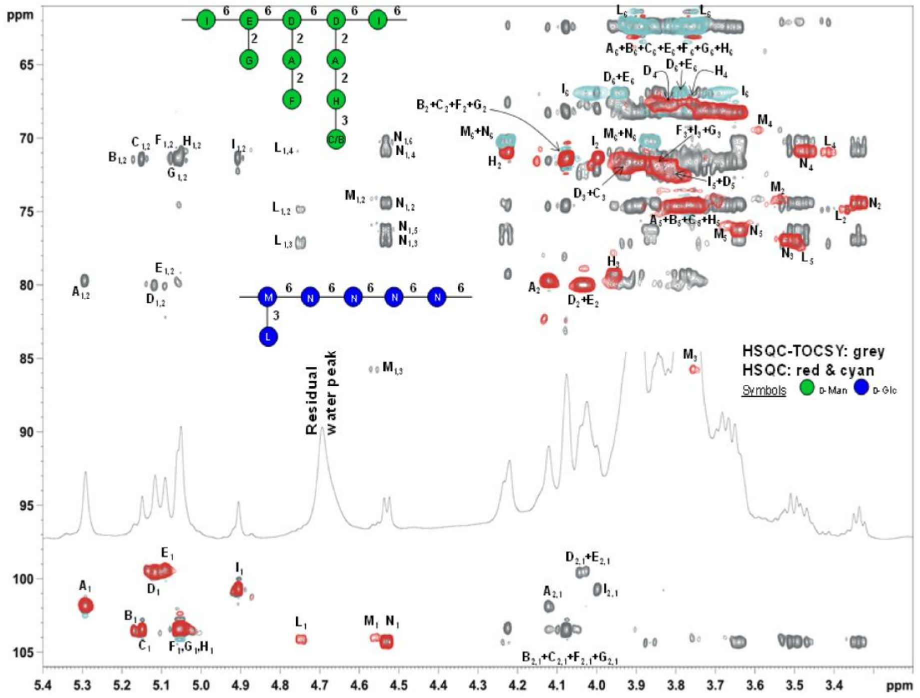

c

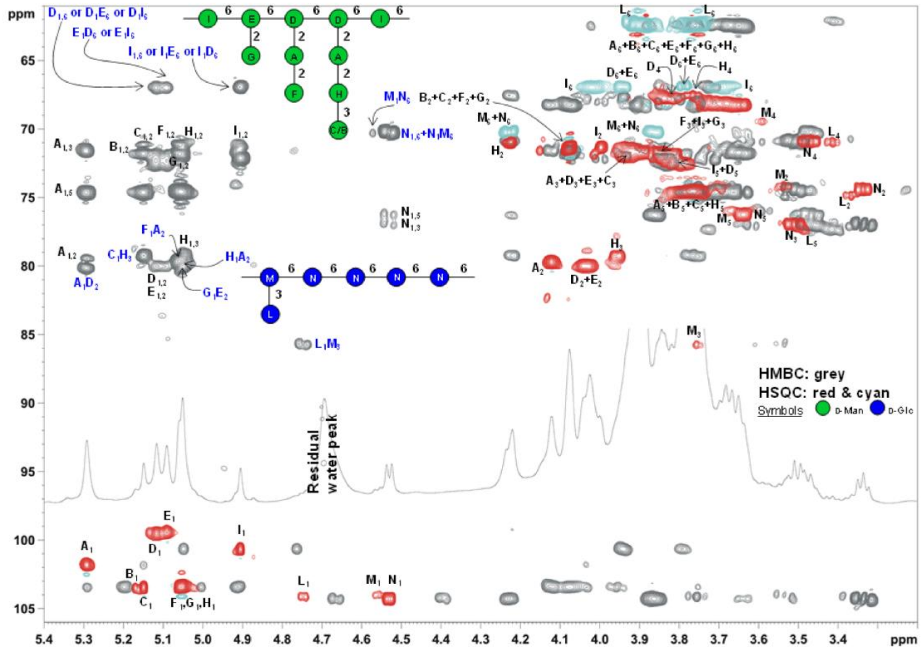

Figure S2. Chemical properties of MGCP. (**a**) Scheme of protocol for polysaccharide purification from yeast extract. As described in detail in Materials and Methods, water soluble polysaccharides were purified through removing insoluble polysaccharides, proteins, lipids from yeast extract. (**b**) Overlap of the HSQC-TOCSY (grey) and HSQC (red & cyan) spectra of the yeast extract, measured at 310K in D_2O at 600 MHz. (**c**) Overlap of the HMBC (grey) and HSQC (red & cyan) spectra of the yeast extract, measured at 310K in D_2O at 600 MHz. The structure of the glycans is drawn by representing the monosaccharides with geometric symbols as specified in the legend inside the panel. (**d**) Composition analyses showing building components of MGCP and proportion of each components.

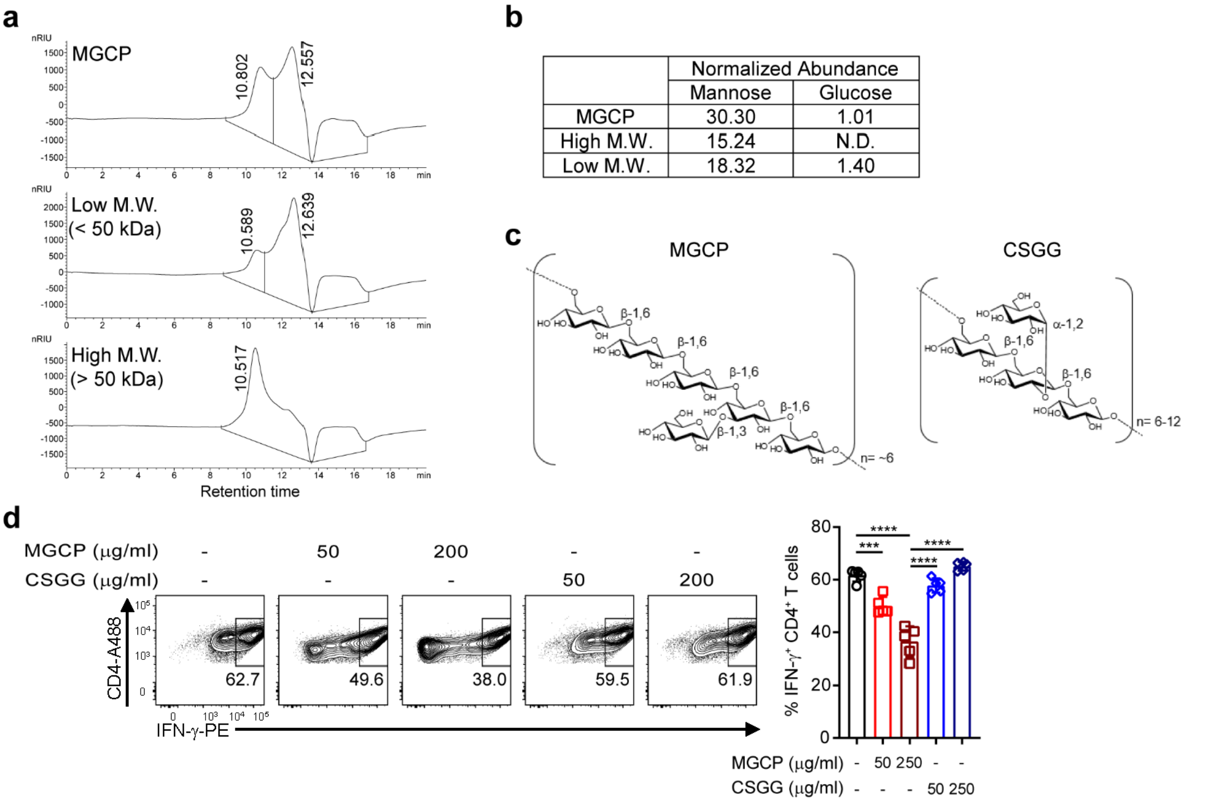

Figure S3. Essential role of β-1,6-glucan for immunological properties of MGCP. (**a**) MGCP was fractionated according to molecular weight by High Performance Liquid Chromatography (HPLC) with 0.3 ml/min fluid speed and D_2O as eluent. Chromatogram profile of MGCP and individual fractions analyzed by refractive index detector are shown. (**b**) Relative abundance of mannose and glucose in MGCP in each fraction. (**c**) Comparison of structure of essential active β-1,6-glucan moiety in MGCP and CSGG. (**d**) Splenic DCs were stimulated with either MGCP or CSGG followed by culture with naïve CD4 T cells in the presence of suboptimal Th1 driving cytokines. Representative FACS plots (left) and frequencies of IFN-γ producing $CD4^+$ T cells (right) are shown. Data is pooled from three independent experiments with 5 samples. Graph shows the mean ± SD. ***p < 0.001, ****p < 0.0001 (Two tailed student's t test). Source data are provided as a Source Data file.

a

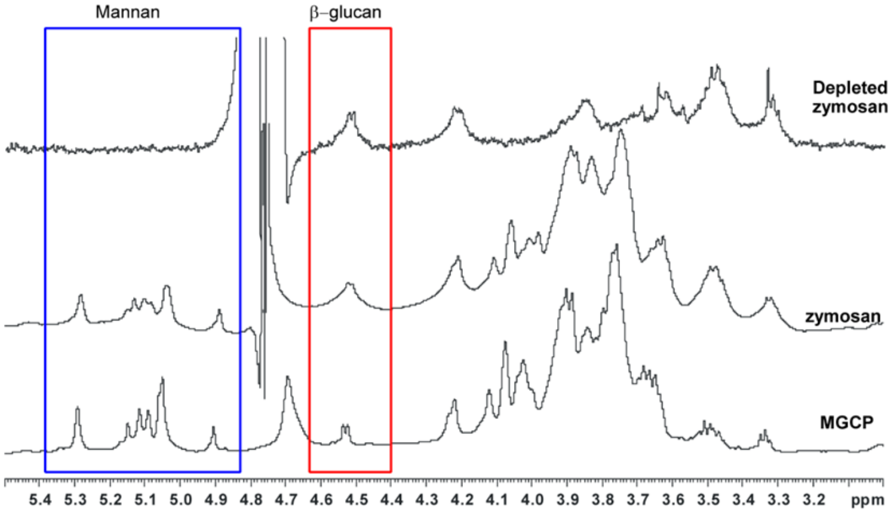

b

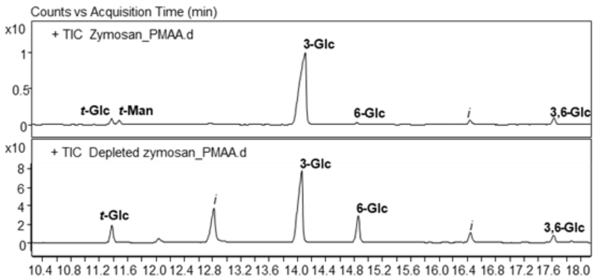

c

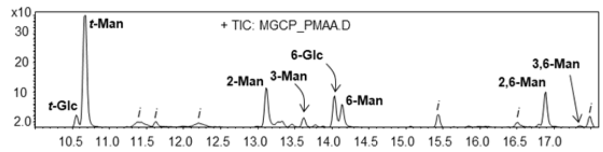

d

	Zymosan	Depleted Zymosan	MGCP
t-Glc	3.5	10.5	24.6
3-Glc	91.2	67.5	0.0
6-Glc	1.2	17.3	71.3
3,6-Glc	4.1	4.7	4.1

Figure S4. Chemical properties of MGCP in comparison with zymosan and depleted zymosan. (**a**) Composition of MGCP, zymosan and depleted zymosan was measured by proton NMR analysis. Proton spectrum of MGCP was recorded at 310K, while all the others are analyzed at 298K. Indicated polysaccharides were dissolved in deuterated water and subjected to NMR analysis. (**b**) Chromatogram of the partially methylated and acetylated alditols of zymosan (top trace) and depleted zymosan (bottom trace). (**c**) MGCP analysis of the partially methylated and acetylated alditols by gas chromatography. (**d**) 3-Linked glucose is distinguished from 6-linked glucose different linked β-glucans are quantified by measuring areas of their representative derivatives of two different zymosans and MGCP.

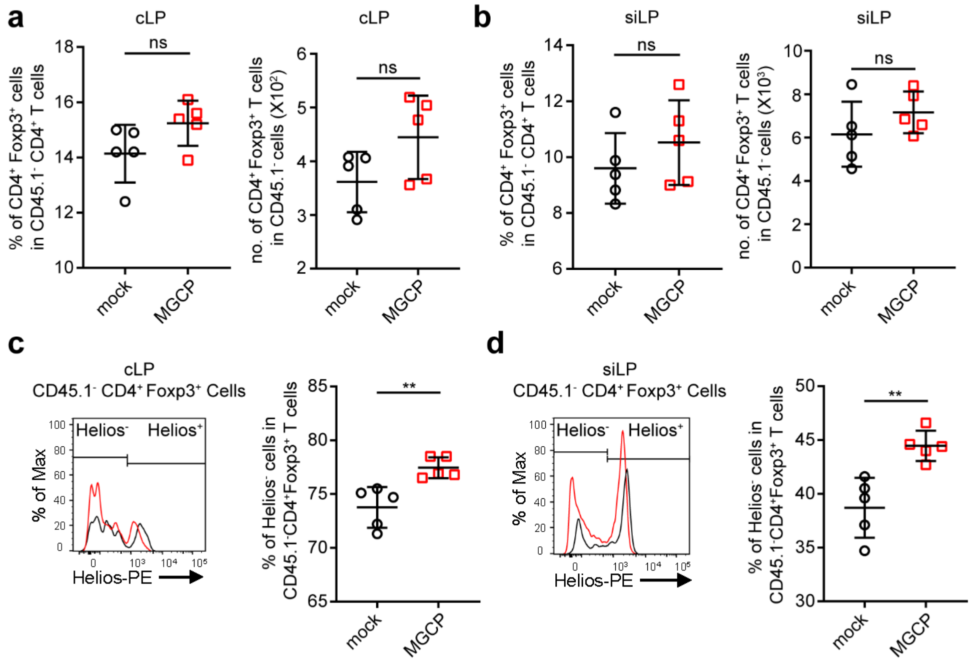

Figure S5. MGCP supplementation enhances iTreg cell differentiation in the intestine. Mice were administered with mock or MGCP daily for 2 weeks and adoptively transferred congenically marked naïve CD 4^+ T cells. Recipient mice were analyzed after one additional week of continuous supplementation of mock or MGCP every day. Intestinal $CD4^+$ T cells were assessed. (**a**, **b**) Frequencies and absolute numbers of recipient derived $CD45.1^-CD4^+Foxp3^+$ T cells in colonic (a) and small intestinal lamina propria (b). (**c**, **d**) Representative FACS plots (left) and frequencies (right) of $Helios^-$ Treg cells in the colon (c) and small intestine (d) of mock and MGCP treated recipient mice. All data are analyzed with pooled data from 2 independent experiments with 5 mice. Each dot represents individual mouse. All graphs show the mean ± SD. $^{**}p < 0.01$ (Two tailed student's t test). ns: not significant. Source data are provided as a Source Data file.

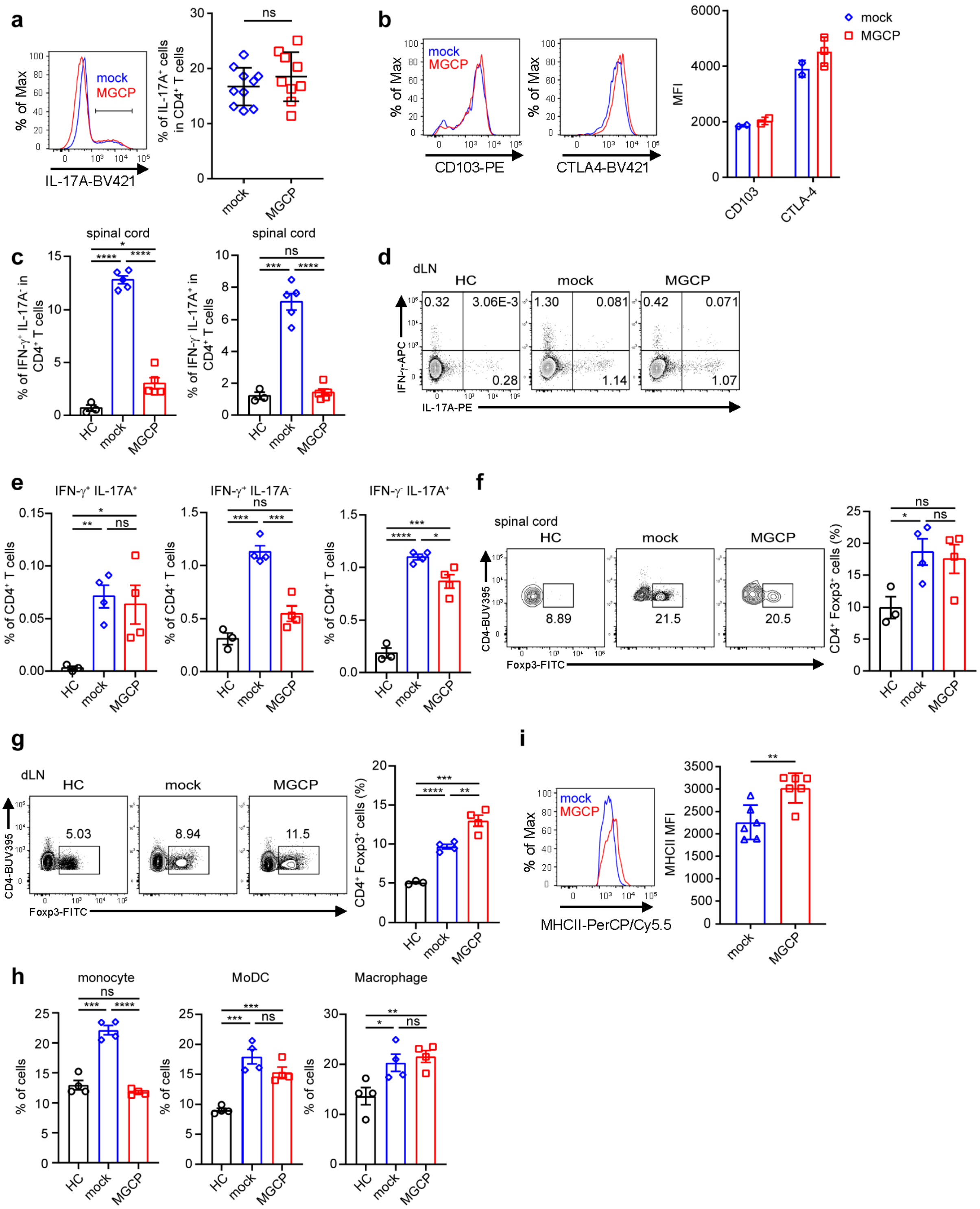
a
% of Max
mock
MGCP
IL-17A-BV421
% of IL-17A+ cells in CD4+ T cells
ns
mock
MGCP
b
% of Max
mock
MGCP
CD103-PE
CTLA4-BV421
MFI
mock
MGCP
CD103
CTLA-4
c
spinal cord
% of IFN-γ+ IL-17A- in CD4+ T cells
% of IFN-γ- IL-17A+ in CD4+ T cells
HC
mock
MGCP
d
dLN
HC
mock
MGCP
0.32
3.06E-3
0.28
1.30
0.081
1.14
0.42
0.071
1.07
IFN-γ-APC
IL-17A-PE
e
IFN-γ+ IL-17A+
IFN-γ+ IL-17A-
IFN-γ- IL-17A+
% of CD4+ T cells
f
spinal cord
HC
mock
MGCP
8.89
21.5
20.5
CD4-BUV395
Foxp3-FITC
CD4+ Foxp3+ cells (%)
g
dLN
HC
mock
MGCP
5.03
8.94
11.5
CD4-BUV395
Foxp3-FITC
CD4+ Foxp3+ cells (%)
i
% of Max
mock
MGCP
MHCII-PerCP/Cy5.5
MHCII MFI
h
monocyte
MoDC
Macrophage
% of cells
HC
mock
MGCP

Figure S6. Impact of MGCP administration in the context of inflammatory diseases. *Rag1* deficient mice were adoptively transferred with naïve $CD4^+$ T cells and fed with mock or MGCP every alternate day. (**a**) Frequency of IL-17A expressing effector $CD4^+$ T cells was determined in colon. Data are analyzed from pooled data of 2 independent experiments with minimum 9 mice. Each dot represents an individual mouse. (**b**) Expression of CD103 and CTLA-4 were assessed in $CD4^+Foxp3^+$ T cells in colon. Mean of CD103 for mock (n=2) and MGCP (n=2) are 1856 and 2030 respectively. Mean of CTLA4 are 3906 and 4526 for mock (n=2) and MGCP (n=3) respectively. (**c-i**) Mice were immunized with MOG_{35-55} and CFA to induce EAE. Immunized mice were injected intraperitoneally with mock or MGCP every day. Frequency (c) of IFN-γ^+ or IL-17A$^+$ CD4 T cells in spinal cord was assessed. Representative flow cytometric plots (d) and frequency (e) of pathogenic cytokine producing effector $CD4^+$ T cells in draining lymph node are shown. Representative flow cytometric plots (left) and frequency (right) of $CD4^+Foxp3^+$ T cells in spinal cord (f) and draining lymph nodes (g) were analyzed. All data are representative of 2 independent experiments with minimum 3 mice (c, e, f, g). (h) Frequency of indicated myeloid cell populations were analyzed in spinal cord. Data is analyzed from 2 independent experiments with 4 mice. Each dot represents an individual mouse. (i) Representative histograms (left) and graphical mean fluorescence intensities (MFI, right) of MHCII on $CD11c^+MHCII^+$ DCs in draining lymph nodes are shown. Data is analyzed from 2 independent experiments with 6 mice. Each dot represents an individual mouse. Flow cytometer plots are representative of two independent experiments with similar results. All graphs show the mean ± SD. *$p < 0.05$, **$p < 0.01$, ***$p < 0.001$, ****$p < 0.0001$ (Two tailed student's t test). ns: not significant. Source data are provided as a Source Data file.

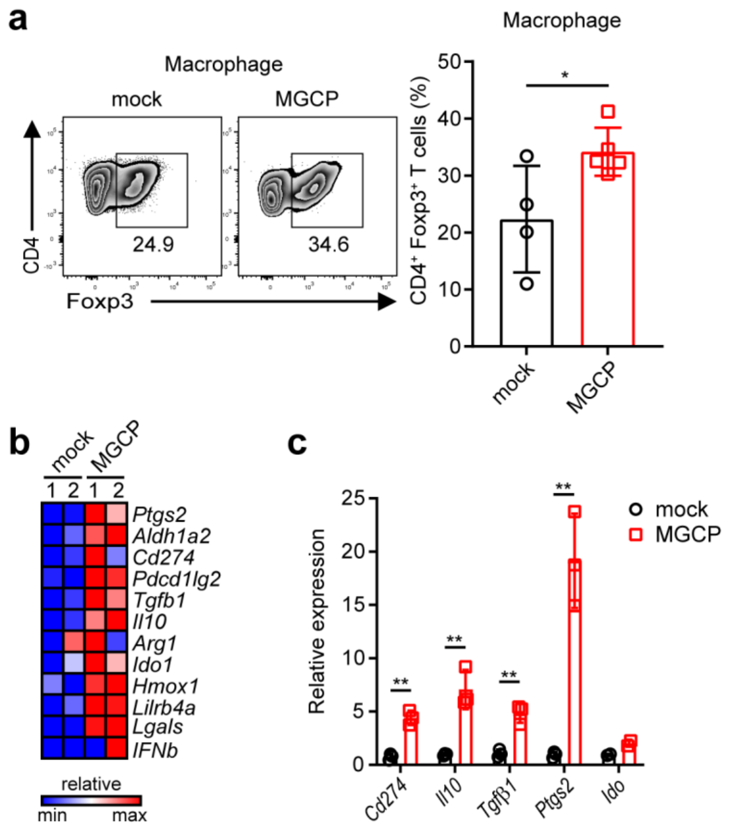

Figure S7. Treg cell differentiation by MGCP is a DC mediated process. (**a**) Macrophage were FACS sorted from lamina propria of small intestine and treated with MGCP or mock, and tested for their capability of inducing Treg cells under suboptimal iTreg inducing conditions. Representative FACS plots (left) and frequencies (right) are shown. Data are representative of two independent experiments with similar results. Data is analyzed from 2 independent experiments with minimum 4 samples. (**b**, **c**) Germ-free mice (n=6) were fed with mock or MGCP daily for 2 weeks. Colonic DCs were sorted by micro beads and total mRNA was purified. (b) Transcriptome was analyzed to compare expression of regulatory associated markers by RNA-seq. (c) Expressions of tolerogenic DC associated markers were verified by RT-qPCR. Data is analyzed with pooled data of two independent experiments with 3 samples (for *Cd274*, *Il10*, *Tgfβ1* and Ptgs2) or 2 samples (*Ido*). All bar graphs show the mean ± SD.

*p < 0.05, **p < 0.01 (Two tailed student's t test). Source data are provided as a Source Data file.

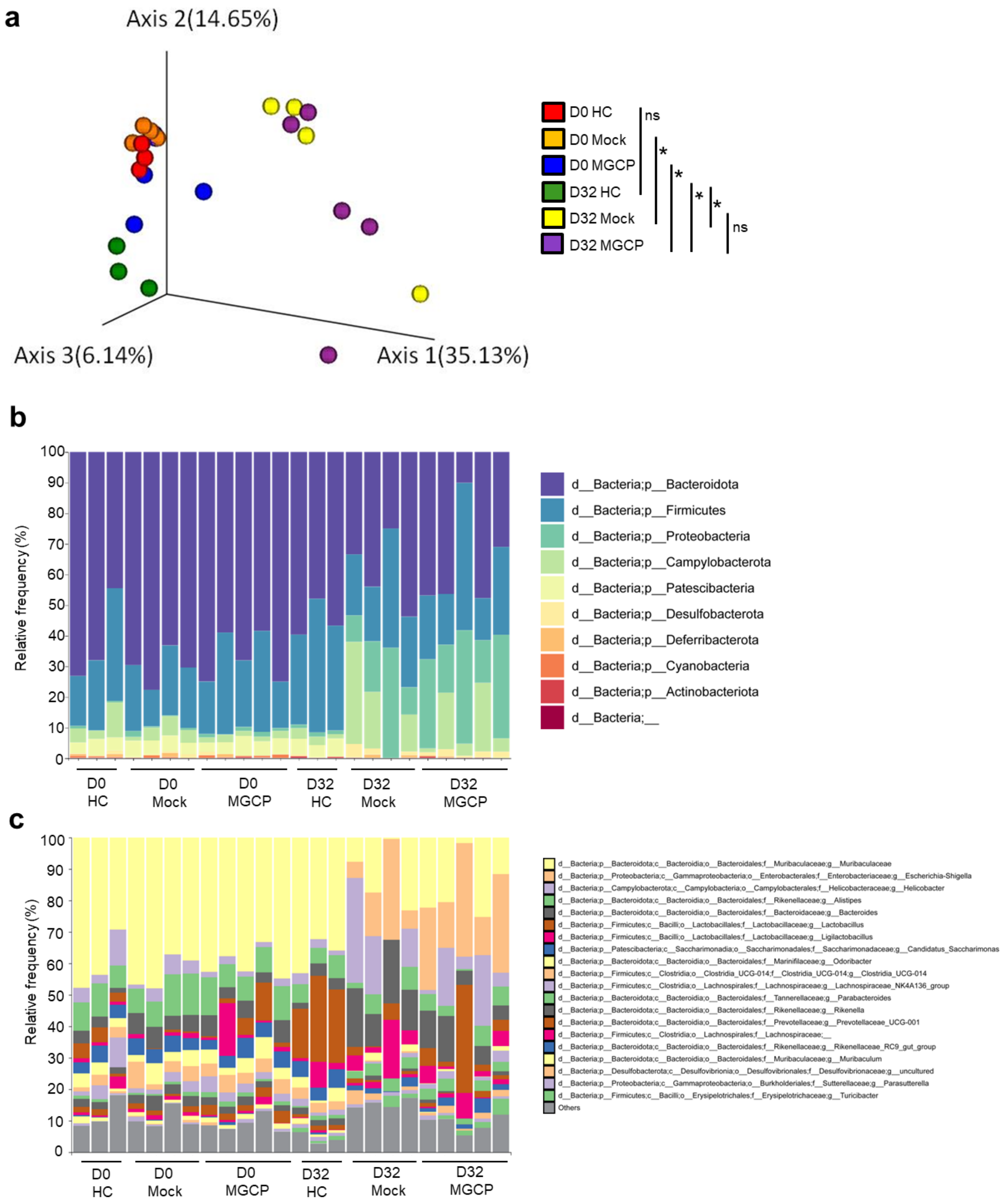

a
Axis 2(14.65%)
Axis 3(6.14%)
Axis 1(35.13%)
D0 HC
D0 Mock
D0 MGCP
D32 HC
D32 Mock
D32 MGCP
ns
*
*
*
*
ns
b
Relative frequency(%)
100
90
80
70
60
50
40
30
20
10
0
D0 HC
D0 Mock
D0 MGCP
D32 HC
D32 Mock
D32 MGCP
d__Bacteria;p__Bacteroidota
d__Bacteria;p__Firmicutes
d__Bacteria;p__Proteobacteria
d__Bacteria;p__Campylobacterota
d__Bacteria;p__Patescibacteria
d__Bacteria;p__Desulfobacterota
d__Bacteria;p__Deferribacterota
d__Bacteria;p__Cyanobacteria
d__Bacteria;p__Actinobacteriota
d__Bacteria;__
c
Relative frequency(%)
100
90
80
70
60
50
40
30
20
10
0
D0 HC
D0 Mock
D0 MGCP
D32 HC
D32 Mock
D32 MGCP
d__Bacteria;p__Bacteroidota;c__Bacteroidia;o__Bacteroidales;f__Muribaculaceae;g__Muribaculaceae
d__Bacteria;p__Proteobacteria;c__Gammaproteobacteria;o__Enterobacterales;f__Enterobacteriaceae;g__Escherichia-Shigella
d__Bacteria;p__Campylobacterota;c__Campylobacteria;o__Campylobacterales;f__Helicobacteraceae;g__Helicobacter
d__Bacteria;p__Bacteroidota;c__Bacteroidia;o__Bacteroidales;f__Rikenellaceae;g__Alistipes
d__Bacteria;p__Bacteroidota;c__Bacteroidia;o__Bacteroidales;f__Bacteroidaceae;g__Bacteroides
d__Bacteria;p__Firmicutes;c__Bacilli;o__Lactobacillales;f__Lactobacillaceae;g__Lactobacillus
d__Bacteria;p__Firmicutes;c__Bacilli;o__Lactobacillales;f__Lactobacillaceae;g__Ligilactobacillus
d__Bacteria;p__Patescibacteria;c__Saccharimonadia;o__Saccharimonadales;f__Saccharimonadaceae;g__Candidatus_Saccharimonas
d__Bacteria;p__Bacteroidota;c__Bacteroidia;o__Bacteroidales;f__Marinifilaceae;g__Odoribacter
d__Bacteria;p__Firmicutes;c__Clostridia;o__Clostridia_UCG-014;f__Clostridia_UCG-014;g__Clostridia_UCG-014
d__Bacteria;p__Firmicutes;c__Clostridia;o__Lachnospirales;f__Lachnospiraceae;g__Lachnospiraceae_NK4A136_group
d__Bacteria;p__Bacteroidota;c__Bacteroidia;o__Bacteroidales;f__Tannerellaceae;g__Parabacteroides
d__Bacteria;p__Bacteroidota;c__Bacteroidia;o__Bacteroidales;f__Rikenellaceae;g__Rikenella
d__Bacteria;p__Bacteroidota;c__Bacteroidia;o__Bacteroidales;f__Prevotellaceae;g__Prevotellaceae_UCG-001
d__Bacteria;p__Firmicutes;c__Clostridia;o__Lachnospirales;f__Lachnospiraceae;__
d__Bacteria;p__Bacteroidota;c__Bacteroidia;o__Bacteroidales;f__Rikenellaceae;g__Rikenellaceae_RC9_gut_group
d__Bacteria;p__Bacteroidota;c__Bacteroidia;o__Bacteroidales;f__Muribaculaceae;g__Muribaculum
d__Bacteria;p__Desulfobacterota;c__Desulfovibrionia;o__Desulfovibrionales;f__Desulfovibrionaceae;g__uncultured
d__Bacteria;p__Proteobacteria;c__Gammaproteobacteria;o__Burkholderiales;f__Sutterellaceae;g__Parasutterella
d__Bacteria;p__Firmicutes;c__Bacilli;o__Erysipelotrichales;f__Erysipelotrichaceae;g__Turicibacter
Others

Figure S8. Effect of MGCP administration on microbiome composition. Naïve $CD4^+$ T cells ($CD4^+Foxp3^-CD44^{lo}CD62L^{hi}$) were adoptively transferred into *Rag1*$^{-/-}$ deficient mice. MGCP, or distilled water as mock, was fed every other day from a day of transferring the cells and continued for the entire course of experiment. Stool samples were collected from individual mouse on the day before cell transfer (Day 0, D0) and at the end of the experiment (Day 32, D32). Microbiome composition were examined by performing 16s rDNA sequencing with bacterial DNA from each stool sample. (**a**) Principal component plot based on Bray-Curtis is shown. *$p < 0.05$ (PERMANOVA), ns: not significant. (**b**) Relative abundance of indicated phyum-level bacterial taxa in feces. (**c**) Stacked bar plots represent the average relative abundance of bacterial taxa in genus level. Each dot or bar represents an individual mouse.

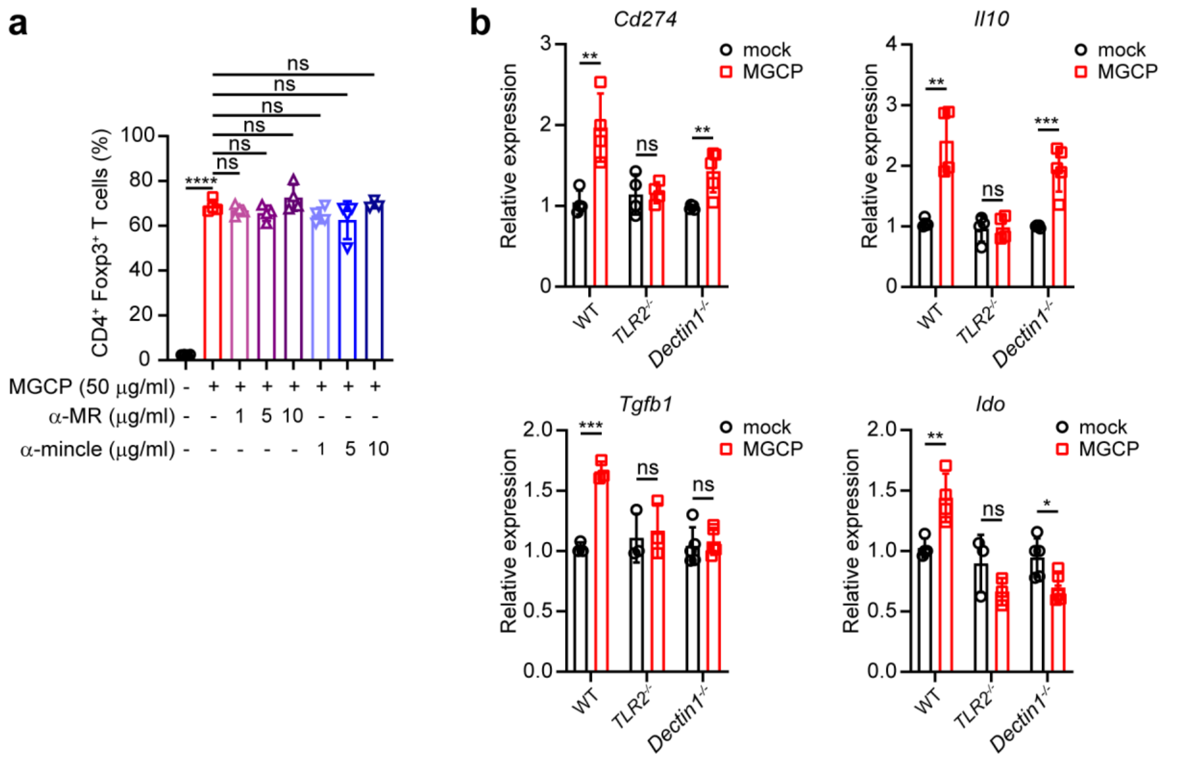

Figure S9. Expression of representative regulatory genes in MGCP treated TLR2$^{-/-}$ and Dectin1$^{-/-}$ DCs. (**a**) Splenic DCs were treated with indicated antagonistic antibodies prior to MGCP stimulation and were cultured with naïve CD4^{+} T cells in the presence of suboptimal amount of Treg driving cytokines. Frequencies of induced CD4^{+}Foxp3^{+} iTreg cells were analyzed. Result is representative data of minimum 3 samples over 2 independent experiments. (**b**) CD11c^{+} DCs from wild-type (WT), TLR2 and Dectin1 deficient mice were stimulated with mock or MGCP for 8 hours. Expressions of indicated regulatory genes were measured by qRT-PCR analyses. Data are presented from 2 independent experiments with minimum 3 samples. All graphs show the mean ± SD. *$p < 0.05$, **$p < 0.01$, ***$p < 0.001$, ****$p < 0.0001$ (Two tailed student's t test). ns: not significant. Source data are provided as a Source Data file.

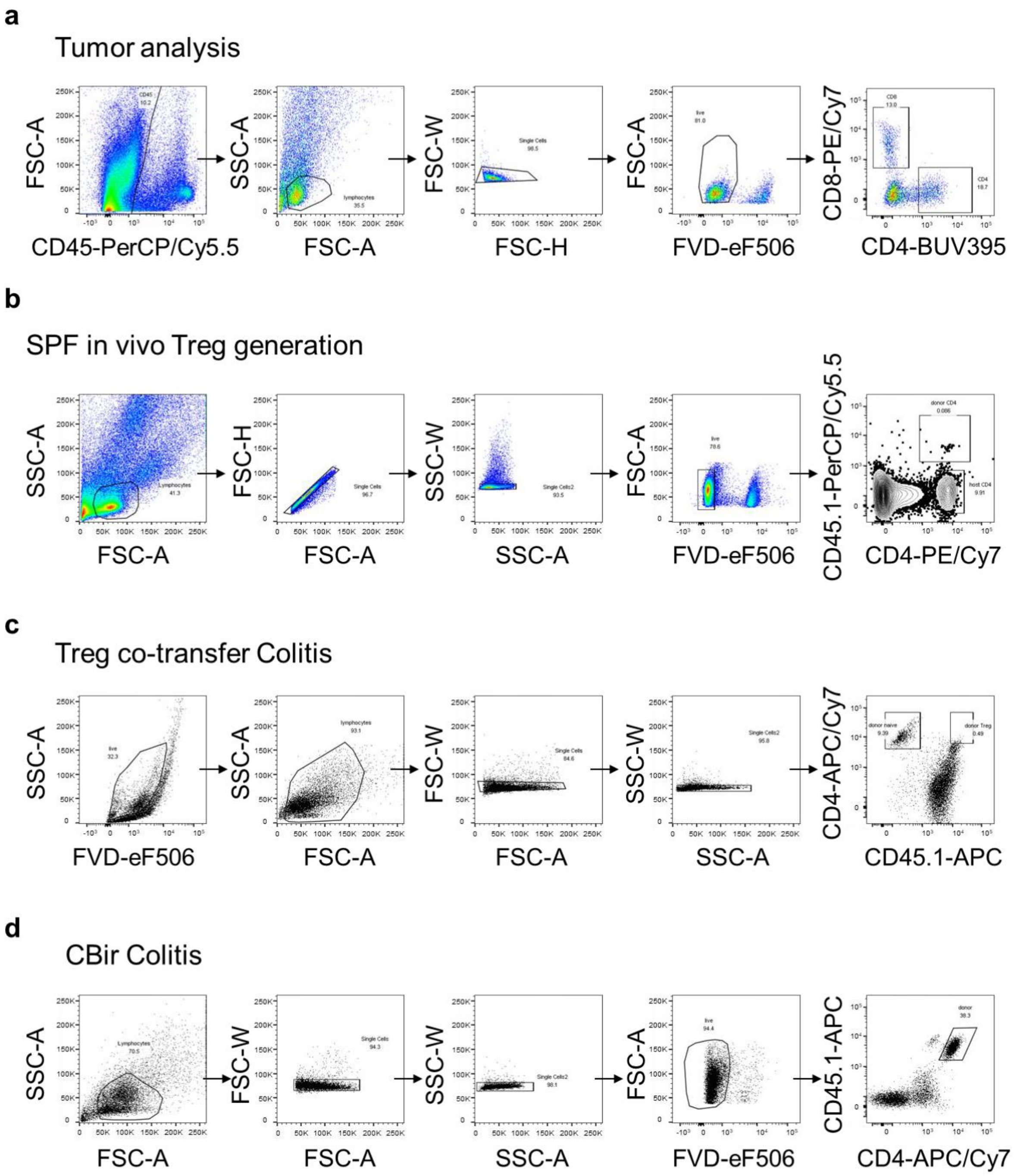
a
Tumor analysis
FSC-A
CD45-PerCP/Cy5.5
SSC-A
FSC-A
FSC-W
FSC-H
FSC-A
FVD-eF506
CD8-PE/Cy7
CD4-BUV395
b
SPF in vivo Treg generation
SSC-A
FSC-A
FSC-H
FSC-A
SSC-W
SSC-A
FSC-A
FVD-eF506
CD45.1-PerCP/Cy5.5
CD4-PE/Cy7
c
Treg co-transfer Colitis
SSC-A
FVD-eF506
SSC-A
FSC-A
FSC-W
FSC-A
SSC-W
SSC-A
CD4-APC/Cy7
CD45.1-APC
d
CBir Colitis
SSC-A
FSC-A
FSC-W
FSC-A
SSC-W
SSC-A
FSC-A
FVD-eF506
CD45.1-APC
CD4-APC/Cy7

Figure S10. Gating strategy for flow cytometric analyses. (**a**) Gating strategy for analysis of tumor infiltrating lymphocytes. (b) Gating strategy of flow cytometric analysis for intestinal lymphocytes with SPF adoptive transfer experiment. (c, d) Gating strategy of colonic lymphocyte analysis for colitis experiments with Treg co-transfer experiment (c) and CBir naïve T cell transfer.

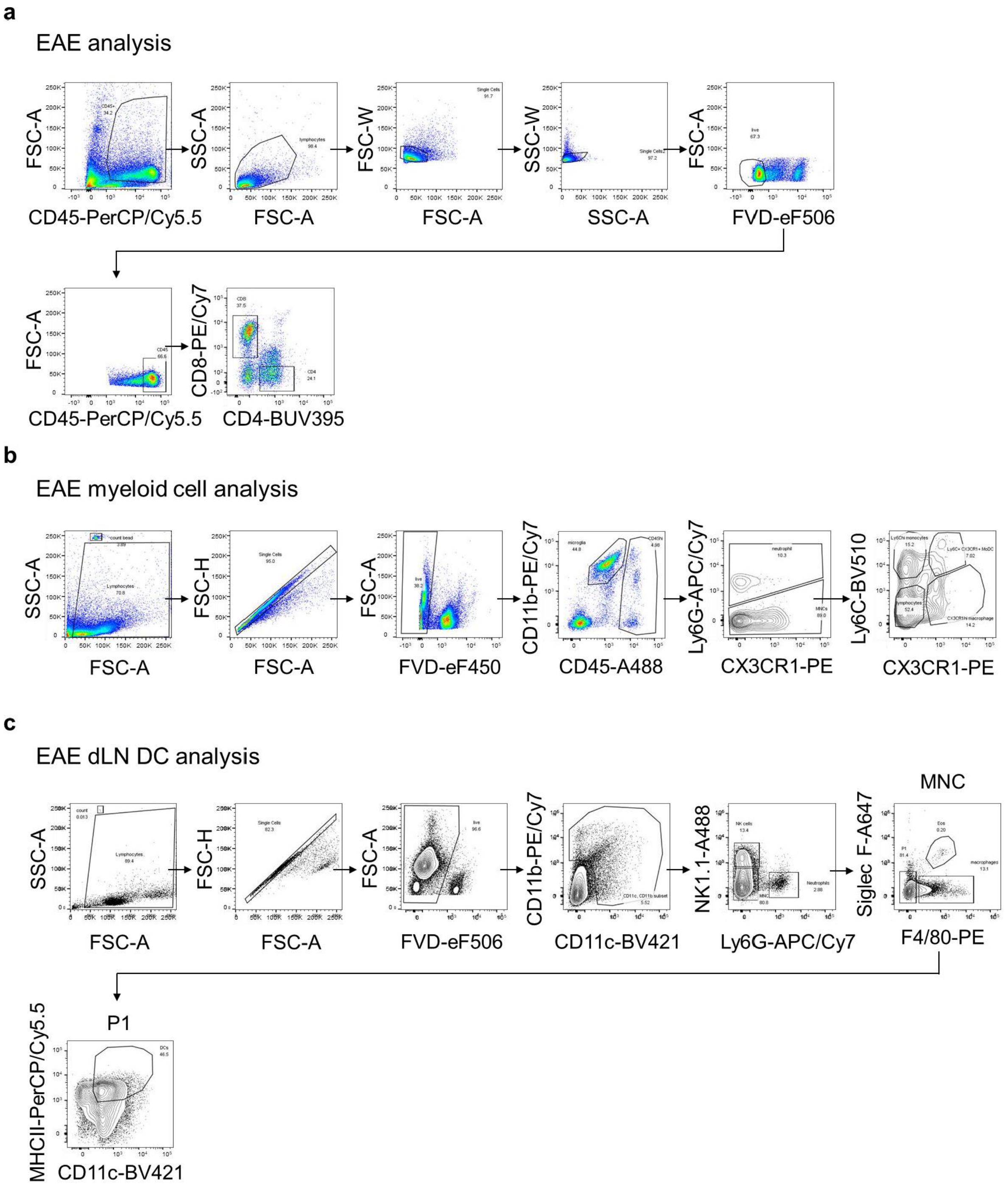
a
EAE analysis
FSC-A
CD45-PerCP/Cy5.5
SSC-A
FSC-A
FSC-W
FSC-A
SSC-W
SSC-A
FSC-A
FVD-eF506
FSC-A
CD45-PerCP/Cy5.5
CD8-PE/Cy7
CD4-BUV395
b
EAE myeloid cell analysis
SSC-A
FSC-A
FSC-H
FSC-A
FSC-A
FVD-eF450
CD11b-PE/Cy7
CD45-A488
Ly6G-APC/Cy7
CX3CR1-PE
Ly6C-BV510
CX3CR1-PE
c
EAE dLN DC analysis
SSC-A
FSC-A
FSC-H
FSC-A
FSC-A
FVD-eF506
CD11b-PE/Cy7
CD11c-BV421
NK1.1-A488
Ly6G-APC/Cy7
MNC
Siglec F-A647
F4/80-PE
P1
MHCII-PerCP/Cy5.5
CD11c-BV421

Figure S11. Gating strategy for flow cytometric analysis of EAE experiments. (**a**) Gating strategy for analysis of CD4 T cells in spinal cord. (b) Gating strategy of flow cytometric analysis of myeloid cell populations in spinal cord. (c) Gating strategy to analyze DCs in draining lymph nodes.

Table S1. ^1H and ^{13}C NMR attribution of MGCP measured in D_2O, at 600 MHz, at 310 K. All the residues are in the pyranose form and have the D absolute configuration; labels are the same used in Figs. S2b and S2c.

Residue	Nucleus	1	2	3	4	5	6
**A**	^1H	5.29	4.12	3.92	3.73	3.77	3.90; 3.76
**α-2-Man**	^{13}C	101.9	79.6	71.4	68.0	74.5	62.3
**B**	^1H	5.17	4.09	3.89	3.65	3.76	3.90; 3.76
**α-*t*-Man**	^{13}C	103.5	71.3	71.7	68.2	74.5	62.3
**C**	^1H	5.15	4.08	3.89	3.65	3.78	3.90; 3.76
**α-*t*-Man**	^{13}C	103.5	71.2	71.6	68.2	74.5	62.3
**D**	^1H	5.12	4.04	3.95	3.83	4.03	3.94; 3.79
**α-2,6-Man**	^{13}C	99.4	80.0	71.9	67.6	72.4	66.8
**E**	^1H	5.09	4.02	3.95	3.83	4.03	3.94; 3.79
**α-2,6-Man**	^{13}C	99.4	79.9	71.9	67.6	72.4	66.8
**F**	^1H	5.06	4.08	3.85	3.65	3.78	3.90; 3.76
**α-*t*-Man**	^{13}C	103.5	71.4	71.8	68.2	74.6	62.3
**G**	^1H	5.05	4.08	3.81	3.69	3.78	3.90; 3.76
**α-*t*-Man**	^{13}C	103.5	71.4	71.8	68.0	74.5	62.3
**H**	^1H	5.05	4.22	3.96	3.77	3.76	3.90; 3.76
**α-3-Man**	^{13}C	103.5	70.8	79.2	67.5	74.5	62.3
**I**	^1H	5.91	4.00	3.84	3.73	ca. 3.8	4.01; 3.69
**α-6-Man**	^{13}C	101.0	71.2	72.1	69.8	72.4	66.9
**L**	^1H	4.75	3.36	3.54	3.41	3.48	3.72; 3.92
**β-*t*-Glc**	^{13}C	103.9	74.7	76.9	70.8	77.2	61.9
**M**	^1H	4.56	3.54	3.75	3.59	3.67	4.23; 3.86
**β-3,6-Glc**	^{13}C	103.9	74.1	85.7	69.3	75.9	70.3
**N**	^1H	4.53	3.33	3.51	3.47	3.64	4.23; 3.86
**β-3-Glc**	^{13}C	104.2	74.4	76.9	70.7	76.1	70.3

Supplementary table 2. List of antibodies used
Flow cytometry

Target	Conjugate	Source	#Catalog	Clone	Dilution factor
CD45	APC	TONBO	20-0451-U100	30-F11	1:500
CD45	PE/Cy7	Biolegend	103114	30-F11	1:500
CD45	PerCP/Cy5.5	Biolegend	103132	30-F11	1:500
CD45	A488	Biolegend	103122	30-F11	1:500
CD45	BV605	Biolegend	103140	30-F11	1:500
CD45.1	PE	Biolegend	110708	A20	1:500
CD45.1	APC	Biolegend	110714	A20	1:500
CD45.1	PerCP/Cy5.5	Biolegend	110728	A20	1:500
CD3	PerCP/Cy5.5	Biolegend	100328	145-2C11	1:500
TCRVa2	APC	Thermo	17-5812-82	B20.1	1:500
CD4	Pacific Blue	Biolegend	100531	RM4-5	1:500
CD4	A488	Biolegend	100529	RM4-5	1:500
CD4	BUV395	BD bioscience	563790	GK1.5	1:500
CD4	APC	Biolegend	100516	RM4-5	1:500
CD4	PE	TONBO	50-0042-U100	RM4-5	1:500
CD4	BV421	Biolegend	100563	RM4-5	1:500
CD4	PE/Cy7	Biolegend	100528	RM4-5	1:500
CD4	APC/Cy7	Biolegend	100526	RM4-5	1:500
CD8a	PE	TONBO	50-0081-U100	53-6.7	1:500
MHCII	PerCP/Cy5.5	Biolegend	107625	M5/114.15.2	1:400
MHCII	FITC	TONBO	35-5321-U100	M5/114.15.2	1:400
CD11c	BV421	Biolegend	117329	N418	1:400
CD11c	PE/Cy7	TONBO	60-0114-U100	N418	1:400
CD11c	APC	Biolegend	117310	N418	1:400
CD11b	PE/Cy7	Biolegend	101216	M1/70	1:400
CD11b	A488	Biolegend	101217	M1/70	1:400
B220	APC	TONBO	20-0452-U100	RA3-6B2	1:400
CD103	PE	Biolegend	121406	2E7	1:200
CD103	BV421	Biolegend	121422	2E7	1:200
CD64	APC	Biolegend	139306	X54-5/7.1	1:100
F4/80	PE	Biolegend	123110	BM8	1:100
F4/80	A488	Biolegend	123120	BM8	1:100
CX3CR1	PE	Biolegend	149006	SA011F11	1:100
NK1.1	A488	Biolegend	108718	PK136	1:200
Ly-6G	APC	Biolegend	127613	1A8	1:200

Continued in next page.

Flow cytometry

Target	Format	Source	#Catalog	Clone	Dilution factor
Ly6G	APC/Cy7	BD bioscience	560600	1A8	1:200
Ly-6G/C (Gr-1)	PE	Biolegend	108408	RB6-8C5	1:200
Ly-6C	BV510	Biolegend	128033	HK1.4	1:200
Nrp1	APC	Thermo	17-3041-82	3DS304M	1:100
Siglec F	A647	BD Bioscience	562680	E50-2440	1:100
CTLA-4 (CD152)	BV421	Biolegend	106312	UC10-4B9	1:100
CD44	APC	Biolegend	103012	IM7	1:400
CD62L	PE	TONBO	50-0621-U100	MEL-14	1:400
Thy-1.1 (CD90.1)	A488	Biolegend	202506	OX-7	1:500
Thy-1.1 (CD90.1)	Pacific Blue	Biolegend	202522	OX-7	1:500
Foxp3	FITC	Thermo	11-5773-82	FJK-16s	1:200
Foxp3	APC	Thermo	17-5773-82	FJK-16s	1:200
Foxp3	PE	Thermo	12-5773-82	FJK-16s	1:200
IFN-g	APC	Thermo	17-7311-81	XMG1.2	1:100
IFN-g	PE/Cy7	Thermo	25-7311-82	XMG1.2	1:100
IFN-g	PE	Thermo	12-7311-82	XMG1.2	1:100
IL-17A	APC	Biolegend	506916	TC11-18H10.1	1:100
IL-17A	PE	Biolegend	506904	TC11-18H10.1	1:100
IL-17A	BV421	Biolegend	506925	TC11-18H10.1	1:100
IL-10	BV421	Biolegend	505021	JES5-16E3	1:100
Helios	PE	Thermo	12-9883-42	22F6	1:100
FVD	eF506	Thermo	65-0866-18		1:1000
FVD	eF450	Thermo	65-0863-18		1:1000

Receptor blocking

Target	Format	Source	#Catalog	Clone
DC-SIGN	purified	abcam	ab13487	120507
Mincle	purified	MBL	D266-3	1B6
CD206(MMR)	purified	Biolegend	321101	15-2

Supplementary table 3. List of primers used
Quantitative reverse transcriptase PCR

Hprt	F	5'-TTA TGG ACA GGA CTG AAA GAC-3’
	R	5'-GCT TTA ATG TAA TCC AGC AGG T-3’
Il-10	F	5'-ATA ACT GCA CCC ACT TCC CA-3’
	R	5'-TCA TTT CCG ATA AGG CTT GG-3’
Tgf-b	F	5'-CTC CCG TGG CTT CTA GTG C-3’
	R	5'-GCC TTA GTT TGG ACA GGA TCT G-3’
Cd274	F	5'-GCT CCA AAG GAC TTG TAC GTG-3’
	R	5'-TGA TCT GAA GGG CAG CAT TTC-3’
Il-27	F	5'-CAC CTC CGC TTT CAG GTG C-3’
	R	5'-AGG TAT AGA GCA GCT GGG GC-3’
Ido	F	5'-GCT TTG CTC TAC CAC ATC CAC-3’
	R	5'-CAG GCG CTG TAA CCT GTG T-3’
Ptgs2	F	5'-TGG CTG CAG AAT TGA AAG CCC T-3’
	R	5'-AAA GGT GCT CGG CTT CCA GTA T-3’
Arg1	F	5'-CTC CAA GCC AAA GTC CTT AGA G-3’
	R	5'-AGG AGC TGT CAT TAG GGA CAT C-3’
Cd40	F	5'-CCT TGC TGA ACT GTG AGG AGA-3’
	R	5'-CTT CGC TTA CAA CGT GTG CT-3’
Cd80	F	5'-ACC CCC AAC ATA ACT GAG TCT-3’
	R	5'-TTC CAA CCA AGA GAA GCG AGG-3’
Cd86	F	5'-TGT TTC CGT GGA GAC GCA AG-3’
	R	5'-CAG CTC ACT CAG GCT TAT GTT TT-3’
