# Supplementary material for: Structural specificities of cell surface β-glucan polysaccharides determine commensal yeast mediated immuno-modulatory activities
Source: Nat Commun. 2021 Jun 14;12:3611. doi: 10.1038/s41467-021-23929-9 (PMC8203763; doi:10.1038/s41467-021-23929-9)
Supplement: Supplementary file 3 — Reporting summary [file 41467_2021_23929_MOESM3_ESM.pdf]

nature research

Corresponding author(s): Dipayan Rudra
Sin-Hyeog Im

Last updated by author(s): 4/21/2021

Reporting Summary

Nature Research wishes to improve the reproducibility of the work that we publish. This form provides structure for consistency and transparency in reporting. For further information on Nature Research policies, see our Editorial Policies and the Editorial Policy Checklist.

Statistics

For all statistical analyses, confirm that the following items are present in the figure legend, table legend, main text, or Methods section.

n/a	Confirmed	
☐	☒	The exact sample size (n) for each experimental group/condition, given as a discrete number and unit of measurement
☐	☒	A statement on whether measurements were taken from distinct samples or whether the same sample was measured repeatedly
☐	☒	The statistical test(s) used AND whether they are one- or two-sided<br>*Only common tests should be described solely by name; describe more complex techniques in the Methods section.*
☐	☒	A description of all covariates tested
☐	☒	A description of any assumptions or corrections, such as tests of normality and adjustment for multiple comparisons
☐	☒	A full description of the statistical parameters including central tendency (e.g. means) or other basic estimates (e.g. regression coefficient) AND variation (e.g. standard deviation) or associated estimates of uncertainty (e.g. confidence intervals)
☐	☒	For null hypothesis testing, the test statistic (e.g. F, t, r) with confidence intervals, effect sizes, degrees of freedom and P value noted<br>*Give P values as exact values whenever suitable.*
☒	☐	For Bayesian analysis, information on the choice of priors and Markov chain Monte Carlo settings
☒	☐	For hierarchical and complex designs, identification of the appropriate level for tests and full reporting of outcomes
☒	☐	Estimates of effect sizes (e.g. Cohen's d, Pearson's r), indicating how they were calculated

Our web collection on statistics for biologists contains articles on many of the points above.

Software and code

Policy information about availability of computer code

Data collection	BD FACSDiva software was used for FACS data collection.
Data analysis	All statistical analyses were done using Graphpad Prism 8. All FACS data analyses were done using Flowjo v10.5.0 (Treestar).

For manuscripts utilizing custom algorithms or software that are central to the research but not yet described in published literature, software must be made available to editors and reviewers. We strongly encourage code deposition in a community repository (e.g. GitHub). See the Nature Research guidelines for submitting code & software for further information.

Data

Policy information about availability of data

All manuscripts must include a data availability statement. This statement should provide the following information, where applicable:

- Accession codes, unique identifiers, or web links for publicly available datasets
- A list of figures that have associated raw data
- A description of any restrictions on data availability

All data needed to evaluate the conclusions in the paper are present in the paper and/or the Supplementary Materials. Additional data related to this paper may be requested from the authors. RNA-seq data was deposited in the Gene Expression Omnibus (NCBI) data repository under accession number GEO: RNA-seq data with splenic DCs: GSE150685. RNA-seq data with colonic DCs: GSE126937.

Field-specific reporting

Please select the one below that is the best fit for your research. If you are not sure, read the appropriate sections before making your selection.

☒ Life sciences ☐ Behavioural & social sciences ☐ Ecological, evolutionary & environmental sciences

For a reference copy of the document with all sections, see nature.com/documents/nr-reporting-summary-flat.pdf

Life sciences study design

All studies must disclose on these points even when the disclosure is negative.

Sample size	No sample-size calculation was performed. At least three mice data either representative or pooled from multiple experiments were used.
Data exclusions	No data was excluded.
Replication	All experiments could be successfully replicated.
Randomization	Age and sex-matched mice were assigned randomly in experimental and control groups.
Blinding	Blinding assessment was performed during histology scoring and weight and clinical score measurement of animals in colitis and EAE experiments. For other experiments, the investigators were not blinded to group allocations during data collection and analysis. This approach is considered standard for these kinds of experiments.

Reporting for specific materials, systems and methods

We require information from authors about some types of materials, experimental systems and methods used in many studies. Here, indicate whether each material, system or method listed is relevant to your study. If you are not sure if a list item applies to your research, read the appropriate section before selecting a response.

Materials & experimental systems

n/a	Involved in the study	
☐	☒	Antibodies
☐	☒	Eukaryotic cell lines
☒	☐	Palaeontology and archaeology
☐	☒	Animals and other organisms
☒	☐	Human research participants
☒	☐	Clinical data
☒	☐	Dual use research of concern

Methods

n/a	Involved in the study	
☒	☐	ChIP-seq
☐	☒	Flow cytometry
☒	☐	MRI-based neuroimaging

Antibodies

Antibodies used	The information is provided in Methods section and Supplementary Table 2. All FVD staining was done with 1:1000 dilution. 1:500 dilution factor was used for CD45(30-F11, Biolegend), CD45.1 (A20, Biolegend), CD3 (145-2C11, Biolegend), TCRVa2 (B20.1, Thermo), CD4 (RM4-5, GK1.5, Biolegend/TONBO and BD bioscience respectively), CD8a (53-6.7, TONBO) and Thy1.1 (OX-7, Biolegend) antibodies. 1:400 dilution was used for MHCII (M5/114.15.2, Biolegend or TONBO), CD11c (N418, Biolegend or TONBO), CD11b (M1/70, Biolegend) and B220 (RA3-6B2, TONBO) antibodies. 1:200 dilutions was used for antibodies with CD103 (2E7, Biolegend), NK1.1 (PK136, Biolegend), Ly6G (1A8, Biolegend or BD Bioscience), Ly6G/C (RB6-8C5, Biolegend), Ly6C (HK1.4, Biolegend) and Foxp3 (FJK-16s, Thermo). 1:100 dilution was used for CD64 (X545/7.1, Biolegend), F4/80 (Bm8, Biolegend), CX3CR1 (SA011F11, Biolegend), Nrp1 (3DS304M, Thermo), SiglecF (E50-2440, BD Bioscience), CTLA-4 (UC10-4B9, Biolegend), CD44 (IM7, Biolegend), CD62L (MEL-14, TONBO), IFN-g (XMG1.2, Thermo), IL-17A (TC11-18H10.1, Biolegend), IL-10 (JES5-16E3, Biolegend) and Helios 22F6, Thermo).
Validation	The specificities of the listed antibodies used for FACS have been validated by the manufacturer by flow cytometry. For DC-SIGN antibody, concentration with 5ug/ml was recommended according to manufacturer's technical data sheet. Blocking antibody for Mincle was used according to manufacturer's recommendation which is 1-10ug/ml for blocking of functional activity. For anti-Mannose receptor antagonistic antibody, we used dose of antibody according to manufacturer's data sheet and reference paper with PMID number 27413759.

Eukaryotic cell lines

Policy information about cell lines

Cell line source(s)	B16-F10 cell lines were purchased from ATCC.

Authentication	The cell line used was not authenticated.
Mycoplasma contamination	The cell line used was not tested for mycoplasma contamination.
Commonly misidentified lines (See ICLAC register)	No commonly misidentified cell line was used.

Animals and other organisms

Policy information about studies involving animals; ARRIVE guidelines recommended for reporting animal research

Laboratory animals	Mice were maintained in the animal facility POSTECH Biotech Center in specific pathogen free condition. All genetic models were in C57BL/6 background. Both male and female mice were used for analyses and quantification.
Wild animals	The study does not involve any wild animal.
Field-collected samples	The study does not involve any sample collected from field.
Ethics oversight	Mice were maintained in the animal facility POSTECH Biotech Center. The POSTECH Institutional Animal Care and Use Committee approved all the experiments.

Note that full information on the approval of the study protocol must also be provided in the manuscript.

Flow Cytometry

Plots

Confirm that:

- [x] The axis labels state the marker and fluorochrome used (e.g. CD4-FITC).
- [x] The axis scales are clearly visible. Include numbers along axes only for bottom left plot of group (a 'group' is an analysis of identical markers).
- [x] All plots are contour plots with outliers or pseudocolor plots.
- [x] A numerical value for number of cells or percentage (with statistics) is provided.

Methodology

Sample preparation	For isolation of lymphocytes from colon, small intestine and spinal cord, tissues were opened longitudinally and rinsed with PBS to remove mucus and feces. Intestines were cut into pieces with 0.5~1 cm and incubated with PBS containing 10 mM EDTA, 20 mM HEPES, 1 mM Sodium pyruvate and 3% of FBS while stirring with magnetic bar for 20 minutes at 37 ℃. Tissue was washed with PBS, then minced followed by incubating in RPMI 1640 media with 3% FBS, 20 mM HEPES, 1 mM Sodium pyruvate, 0.5 mg/ml of Collageanse D (Roche) and DNase I (Sigma-Aldrich) for 45 minutes at 37 ℃. Tissue was incubated for additional 5 minutes in the presence of 10 mM EDTA. Supernatant was filtered with 100 µm cell strainer and transferred into chilled PBS to remove remaining enzymes and EDTA. Cells were loaded onto 40% and 75% PercollTM (GE Healthcare) gradient. Lymphocytes were harvested from interface percoll gradient layer and washed with DMEM media supplemented with 1% FBS, 1% penicillin/streptomycin. For analysis of cytokine, cells were stimulated with PMA (Calbiochem) and Ionomycin (Calbiochem) in the presence of Golgistop (BD Biosciences) for 4-5 hours in complete RPMI media containing 10% FBS, 1% penicillin/streptomycin, 2 mM L-glutamine, 1 mM Sodium pyruvate, non-essential amino acids and 0.1% β-ME (v/v) at 37℃. Cells were stained for analysis with flow cytometry following manufacturer's protocol.
Instrument	LSRII (BD BioSciences), Astrios, Beckman Coulter
Software	BD FACSDiva software (LSR II) was used to collect flow cytometry data. Flowjo v10.5.0 (Tree Star) was used to analyze flow cytometry data.
Cell population abundance	The purities of sorted cells were more than 98%.
Gating strategy	For majority of the experiments FSC-A vs. SSC-A gates of the starting cell population were used first, followed by singlet cell identification using appropriate FSC-H, FSC-A or FSC-H, FSC-A and SSC-W, SSC-A gating. Following this, positive populations were determined by the specific antibodies. All the cases FVD gating was done to select out dead cells. Also in case of tumor samples and EAE samples, CD45 staining was done to first gate on lymphocytes and FVD gating was done for the fist gating in case of colitis sample analysis.

- [x] Tick this box to confirm that a figure exemplifying the gating strategy is provided in the Supplementary Information.
